# Potential of artificial intelligence in the diagnosis and treatment of vertebral compression fractures: A 20-year bibliometric analysis (2004–2023)

**DOI:** 10.1097/MD.0000000000044876

**Published:** 2025-10-03

**Authors:** Peng Qiu, Dong-Xia Chen, Xue-Feng Ma, Hang Ren

**Affiliations:** a Shenzhen Pingle Orthopedic Hospital (Shenzhen Pingshan Traditional Chinese Medicine Hospital), Shenzhen, China.

**Keywords:** artificial intelligence, bibliometrics, vertebral compression fractures

## Abstract

**Background:**

Vertebral compression fractures (VCF) are a common cause of pain and disability, particularly in the aging population. Although artificial intelligence (AI) has shown promise across various medical domains, its application in VCF diagnosis and treatment remains fragmented. A comprehensive understanding of the research trends and key contributors to this field is lacking.

**Objective:**

This study aimed to map the knowledge landscape of AI applications in VCF through bibliometric analysis, identifying temporal patterns, intellectual hotspots, and influential contributors to guide future research.

**Methods:**

A total of 462 English-language articles published between 2004 and 2023 were retrieved from the Web of Science Core Collection. CiteSpace 6.2.R6 was used to perform the co-authorship, keyword co-occurrence, citation burst, and clustering analyses. Parameters such as time-slicing, g-index (*k* = 50), and pathfinder network scaling were applied. The key metrics included publication trends, keyword bursts, and centrality scores. Statistical trends were visualized to identify the developmental inflection points and thematic shifts.

**Results:**

The number of publications increased modestly until 2018, followed by a notable surge in 2019, which marked the rapid integration of AI-intensive learning into VCF research. Keyword analysis revealed a thematic evolution from traditional procedures (e.g., vertebroplasty) to AI-driven diagnostics and robotic-assisted interventions. “Deep learning” exhibited the strongest citation burst since 2019. Influential authors, such as Bizhan Aarabi, and institutions in the United States and China were prominent, with SPINE identified as the most frequently cited journal.

**Conclusion:**

AI technologies, especially deep learning and robot-assisted surgery, have become transformative tools in the VCF domain, enhancing diagnostic accuracy and treatment precision. This bibliometric analysis reveals a shift toward technology-driven research paradigms and highlights the critical actors and trends shaping the field. Ongoing interdisciplinary collaboration and clinical validation are essential to fully realize AI’s potential of AI in orthopedic care and improve patient outcomes.

## 1. Introduction

Vertebral compression fractures (VCF) refer to fractures of the vertebral body caused by external compressive forces, often resulting in back pain, diminished stature, and kyphosis, with severe cases potentially affecting neurological functions.^[1,2]^ Such fractures are predominantly observed in the elderly, particularly those with osteoporosis, although they may occur at any age due to trauma or falls.^[3–5]^ Epidemiological studies indicate a significant prevalence rate of VCF, reaching up to 12.63%,^[6]^ and considerably increasing the risk of mortality.^[7,8]^ Early diagnosis before onset and meticulous management post-diagnosis are paramount in rehabilitation strategies for VCF.^[9–11]^

Artificial intelligence (AI), a burgeoning field of technological science, explores and develops theories, methods, technologies, and application systems to simulate, augment, and expand human intelligence, thereby demonstrating substantial advantages in the medical realm.^[12,13]^ AI algorithms efficiently process and analyze medical imagery, such as X-rays, CT scans, and MRIs, enabling the rapid and accurate detection of early indicators of VCF.^[14]^ Medical professionals can leverage AI to analyze patients’ medical histories, lifestyle habits, and genetic data, aiding in the formulation of personalized treatment plans.^[15]^ Furthermore, the utilization of AI technologies in monitoring patients’ health status and recovery progression through wearable devices and mobile applications enhances adherence to treatment plans and allows for timely adjustments,^[16]^ offering an efficient, personalized, and forward-looking solution in the application of AI for VCF management.^[17,18]^

As the advantages of AI in managing VCF have become progressively apparent, a surge of research evidence at various levels continues to emerge. This development underscores the urgent need for detailed and comprehensive literature studies to thoroughly summarize and analyze swiftly appearing research evidence, thereby guiding current hotspots and future research trends. To date, several meta-analyses have been conducted to evaluate the levels of evidence, such as the study by Wang et al,^[19]^ which included 13 articles involving 1094 patients, demonstrating AI’s potential of AI in assisting physicians with percutaneous kyphoplasty and percutaneous vertebroplasty for VCF patients. Additional meta-analyses have independently confirmed AI’s assistance of AI in intraoperative procedures for percutaneous kyphoplasty and percutaneous vertebroplasty.^[20,21]^ Moreover, a meta-analysis by Chen et al,^[22]^ encompassing 12 studies involving 1042 cases, revealed AI’s capability in aiding robotic minimally invasive surgery. Collectively, these studies provide a commendable summary of the application of AI in VCF management. While prior meta-analyses focused on evaluating the effectiveness of specific AI applications, they fell short in capturing the broader structure and evolution of the research field. Key questions remain, such as how the field has developed over time, what its main intellectual foundations are, and who the leading contributors are. Bibliometric analysis addresses this gap by offering a comprehensive “big-picture” view of the knowledge landscape. Tools such as CiteSpace excel at uncovering research trends, influential authors, and collaboration networks, making them especially useful for tracking scientific progress in rapidly evolving areas, such as VCF management.^[23,24]^

To achieve this, our study was guided by the following research questions: what are the temporal evolution, core intellectual themes, and emerging trends in the application of AI to VCF research? Who are the key players (authors, institutions, countries) and what does their collaboration network reveal about the global research landscape in this domain? Despite the growing interest in AI applications for VCF, there is a notable lack of comprehensive bibliometric analyses (BA) in this field. This study aims to fill this gap by conducting a thorough search of publications on AI applications in VCF over the past 20 years. We will objectively visualize bibliometric data regarding trends in annual publication volume and clustering of core keywords, authors, countries, institutions, and journal citation information, thus exploring current research focal points and predicting future directions in this area.

## 2. Materials and methods

### 2.1. Data sources

This study conducted a comprehensive search for publications related to the application of AI in VCF within the Web of Science Core Collection (WoSCC). WoSCC, a comprehensive database of citation information,^[25,26]^ offers unique advantages over other databases (such as PubMed and Cochrane) by allowing direct computation of various bibliometric indicators and presenting a holistic view of core publications in the field without the need for integration across multiple databases.^[27,28]^ For consistency and ease of comparison and validation with similar studies, this database was chosen as the primary source of data.

Although PubMed is widely recognized and utilized in the medical field, it primarily focuses on biomedical literature and may not encompass the full range of publications relevant to our study on AI applications in VCF. WoSCC includes a broader array of journals, encompassing multidisciplinary research that aligns more closely with our focus on BA in this context. Additionally, using WoSCC ensures consistency and ease of comparison with similar studies, reinforcing the validity of our findings.

### 2.2. Search strategy

**Figure 1.**
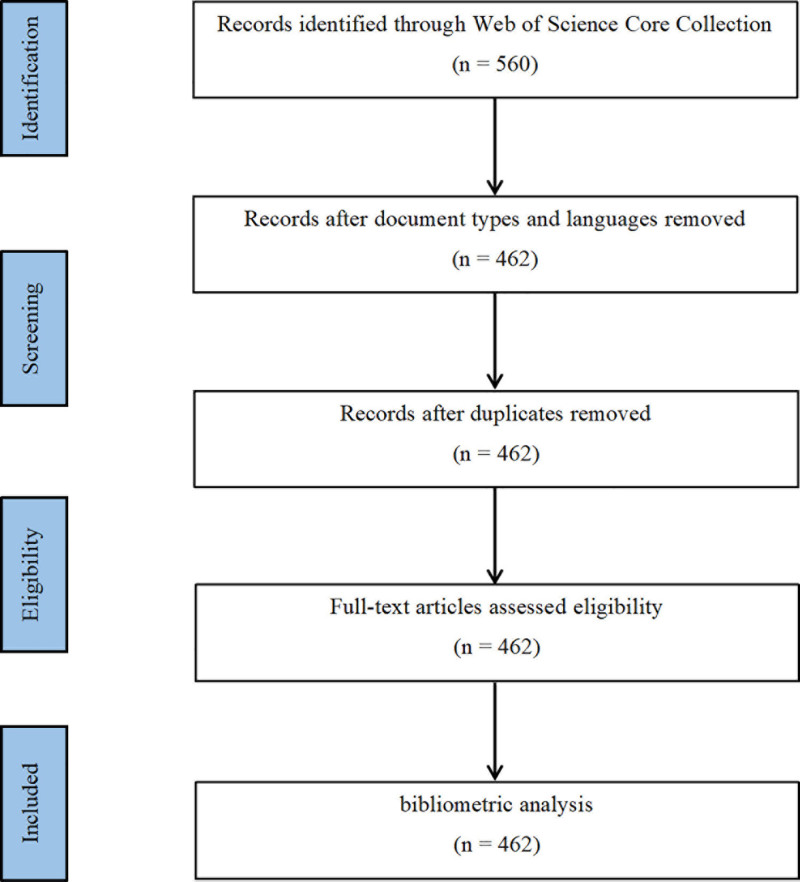
Map of literature screening process related to AI for VCF. AI = artificial intelligence, VCF = vertebral compression fractures.

We filtered publications from January 1, 2004, to December 31, 2023, focusing on keywords related to “Artificial Intelligence” and “Vertebral Compression Fracture,” and formulated a comprehensive search strategy. The search strategy and publication filtering process are shown in Table 1 and Figure 1, culminating in the inclusion of 462 publications. To ensure the comprehensiveness of the data, there were no restrictions on the country of publication, while the language was specified as “English” and the study type as “article.”^[29]^ To ensure consistency in terminology, ease of international comparison, and broader academic visibility, only English-language articles were included, which is a common practice in bibliometric studies.^[30]^

**Table 1:** The topic search query Web of Science Core Collection < January 1, 2004 to December 31, 2023>.

Set	Results	Search query
#1	7443	TS=((Spinal Compression Fractures) OR (Vertebral Compression Fractures) OR (Compression Fractures of the Spine))
#2	3725,377	TS=((Artificial Intelligence) OR (AI) OR (Machine Learning) OR (ML) OR (Deep Learning) OR (Neural Networks) OR (Supervised Learning) OR (Unsupervised Learning) OR (Reinforcement Learning) OR (Natural Language Processing) OR (NLP) OR (Computer Vision) OR (Pattern Recognition) OR (Predictive Analytics) OR (Data Mining) OR (Algorithm) OR (Cognitive Computing))
#3	560	#1 AND #2
#4	462	#3 AND Article (Document Types) AND English (Languages)

### 2.3. Analysis tool

CiteSpace 6.2.R6 software (Professor Chaomei Chen’s Team, Philadelphia) was employed as a visualization analysis tool for this study.^[23,24]^ Renowned for its visualization capabilities, advanced algorithm configurations, and clustering functionalities, CiteSpace has emerged as one of the most widely used tools in the field of BA. Conducting a BA using CiteSpace typically involves several key steps.

1. Data collection: a comprehensive search was conducted in the WoSCC database using the keywords detailed in Table 1, focusing on publications from January 1, 2004, to December 31, 2023. The option to export “Full Record and Cited References” was selected, and data was exported in the “download.txt” format compatible with CiteSpace 6.2. R6.
2. Data import: the collected data were imported into CiteSpace 6.2. R6 software, followed by deduplication and the necessary corrections to ensure data accuracy.
3. Parameter settings: key parameters were set for analysis. The time slice was configured to “1 year” to facilitate yearly trend analysis, allowing for a detailed observation of research developments over time. The g-index *k* value was set to 50, which balances the influence of highly cited articles with those of emerging significance, thus enabling a comprehensive view of both established and novel contributions. Additionally, the “pathfinder network scaling algorithm” was selected to enhance the clarity of the visualizations. This algorithm simplifies complex networks by retaining the most significant connections while reducing visual clutter, thereby allowing for a more effective interpretation of the cooperative relationships among authors, institutions, and research themes.^[31]^
4. Data analysis: with the established parameters, the data were analyzed to cluster information on authors, countries, academic institutions, and cited journals. The visualization of cooperation networks was adjusted to optimally display core information in a reader-friendly manner, facilitating a clear understanding of the research landscape. A citation burstness analysis was used to detect references or keywords with a sudden increase in citations over a specific period, which often indicates emerging topics or breakthroughs. This method is based on the co-citation and co-occurrence networks in CiteSpace. A burst is identified when the growth rate of citations exceeds the set thresholds and lasts for the minimum duration. Strength refers to the intensity of the citation increase for a given keyword or reference during a specific period. This metric helps to identify the top 20 keywords that rapidly gained influence over a Pathfinder network scaling algorithm for a short period.

## 3. Results

### 3.1. Analysis of annual publications and trends

**Figure 2.**
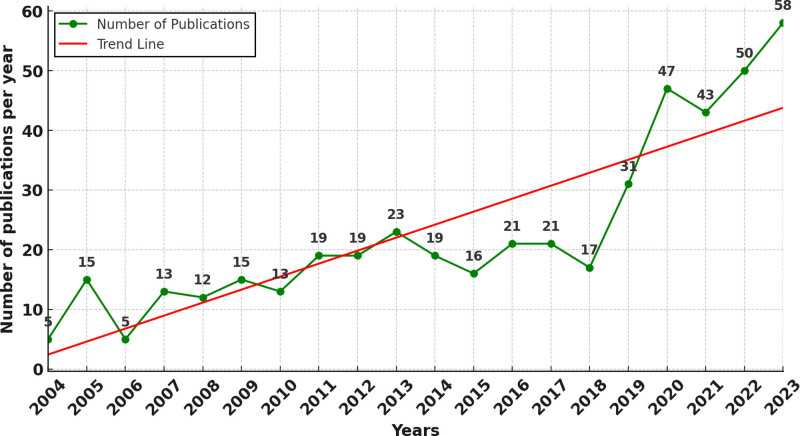
Map of annual publications related to AI for VCF. *Note*: The left vertical axis indicates the annual publication counts, the green dots represent the number of articles published each year, and the red line depicts the average trend of publication during this period. AI = artificial intelligence, VCF = vertebral compression fractures.

Figure 2 depicts a line graph of the temporal changes and growth trends in the number of publications on the application of AI in the VCF domain. Between 2004 and 2018, modest growth in the number of publications was observed. However, a significant increase commenced in 2019, with the growth rate reaching 82.35%, and this high rate of increment was maintained in subsequent years. This indicates that 2019 can be considered a pivotal point, marking the period when AI began to be widely applied and accepted in the VCF field, despite the slower growth in previous years. A study published in the Medical Image Analysis journal (with an impact factor of 10.9) on an iterative fully convolutional neural network for automatic vertebrae segmentation and identification, known for its advanced, fast, and flexible algorithm, was widely disseminated in the AI application to the VCF domain, being cited 144 times.^[32]^ This has significantly propelled the development of this field.

### 3.2. Analysis of keywords

**Figure 3.**
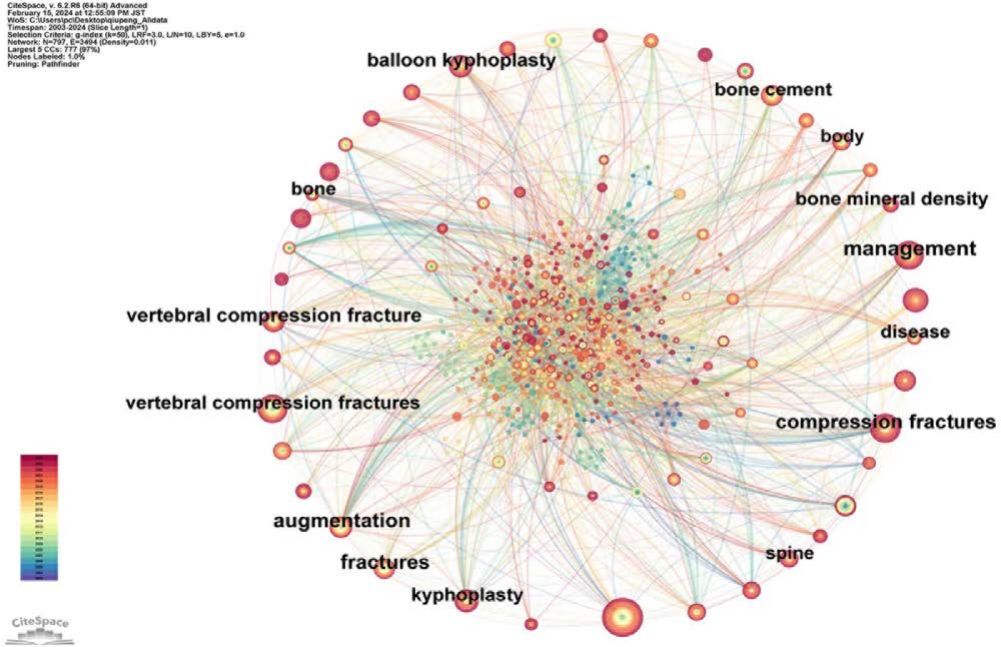
Map of keywords related to AI for VCF. *Note*: Node sizes increase with keyword frequency, and the lines connect 2 keywords co-cited in one study. AI = artificial intelligence, VCF = vertebral compression fractures.

Figure 3 shows a keyword network diagram constructed from 796 keywords and 3460 connections. The top 10 most frequently occurring keywords are listed in Table 2 in descending order: percutaneous vertebroplasty (115 occurrences), compression fractures (83 occurrences), management (61 occurrences), vertebral compression fractures (61 occurrences), balloon kyphoplasty (53 occurrences), vertebroplasty (51 occurrences), kyphoplasty (48 occurrences), risk (38 occurrences), augmentation (31 occurrences), and bone cement (30 occurrences). Among these, the keywords with higher centrality, denoted by a purple ring, included management (0.2), bone mineral density (0.14), vertebral compression fractures (0.12), augmentation (0.12), compression fractures (0.11), and fractures (0.1).

**Table 2:** Top 10 frequency and centrality of keywords related to artificial intelligence for vertebral compression fractures.

Rank	Frequency	Keywords	Rank	Centrality	Keywords
1	115	Percutaneous vertebroplasty	1	0.2	Management
2	83	Compression fractures	2	0.14	Bone mineral density
3	61	Management	3	0.12	Vertebral compression fractures
4	61	Vertebral compression fractures	4	0.12	Augmentation
5	53	Balloon kyphoplasty	5	0.11	Compression fractures
6	51	Vertebroplasty	6	0.1	Fractures
7	48	kyphoplasty	7	0.09	Vertebral compression fracture
8	38	Risk	8	0.08	Osteoporosis
9	31	Augmentation	9	0.08	disease
10	30	Bone cement	10	0.08	Cancellous bone

**Figure 4.**
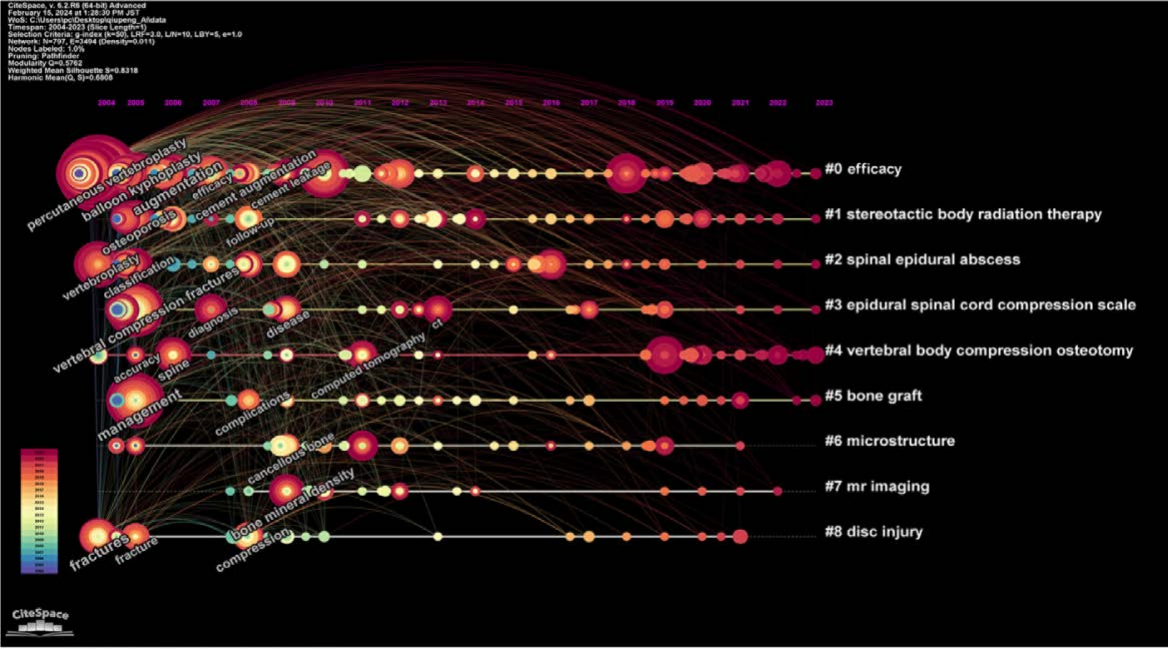
Map of keywords timeline related to AI for VCF. *Note*: A timeline diagram illustrating the relationship between these keywords and their evolution over time: node sizes increase with keyword frequency. AI = artificial intelligence, VCF = vertebral compression fractures.

Through cluster analysis (Fig. 4), we identified 9 core clusters: #0 focusing primarily on percutaneous vertebroplasty and osteoporotic vertebral compression fracture, #1 on bone metastases and spinal metastases, #2 on osteoporosis in postmenopausal women, #3 on deep learning (DL) and imaging data, #4 on various outcome measures, #5 on pediatric VCF, #6 on finite element method and biomechanical data, #7 on magnetic resonance imaging and neuroscientific modulation, and #8 on clinical diagnosis and treatment strategies.

**Figure 5.**
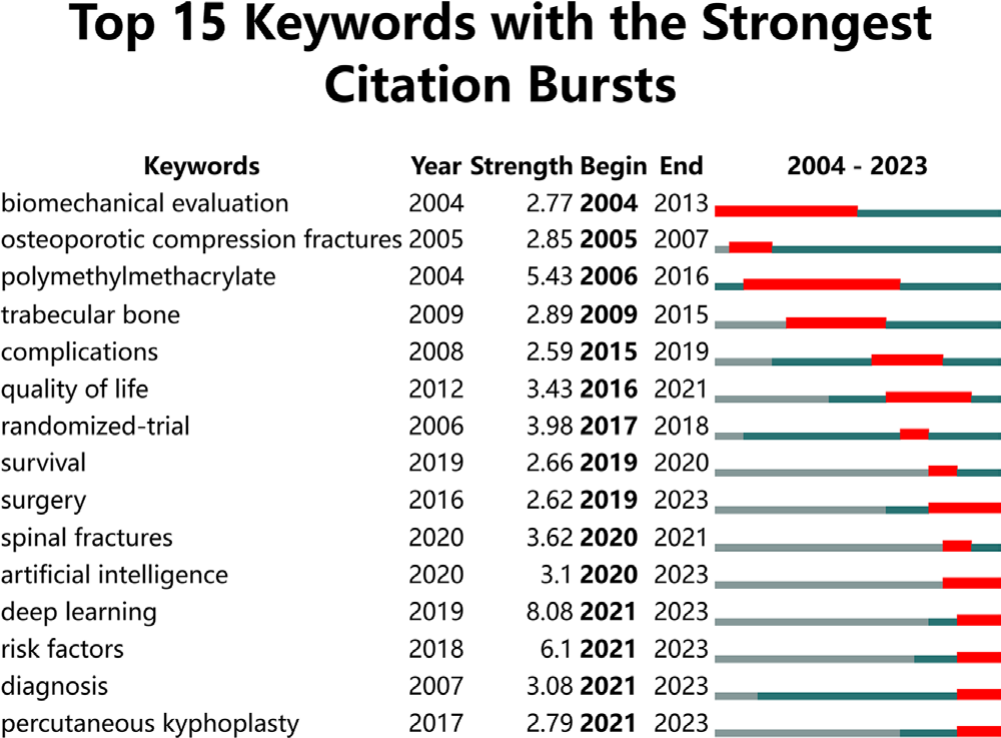
Top 15 keywords with the strongest citation bursts. *Note*: The term “strength” refers to the connection intensity between 2 nodes as determined by the software.

Additionally, we extracted information on the keywords using Strongest Citation Bursts (Fig. 5). This analysis highlights the keywords that experienced significant increases in citation frequency over time, indicating emerging trends and focal points of research interest. Notably, “DL” in 2019 had the highest burst strength (8.08), which signifies a substantial surge in interest and application of this technology in the field of VCF. This marked increase suggests that researchers have begun to recognize the potential of DL algorithms to enhance diagnostic accuracy and treatment efficacy in VCF around this time. This aligns with the 2019 publication surge (Fig. 2), suggesting that the maturation of DL technologies has served as a key catalyst driving the rapid growth of research in this field.

### 3.3. Analysis of authors

**Figure 6.**
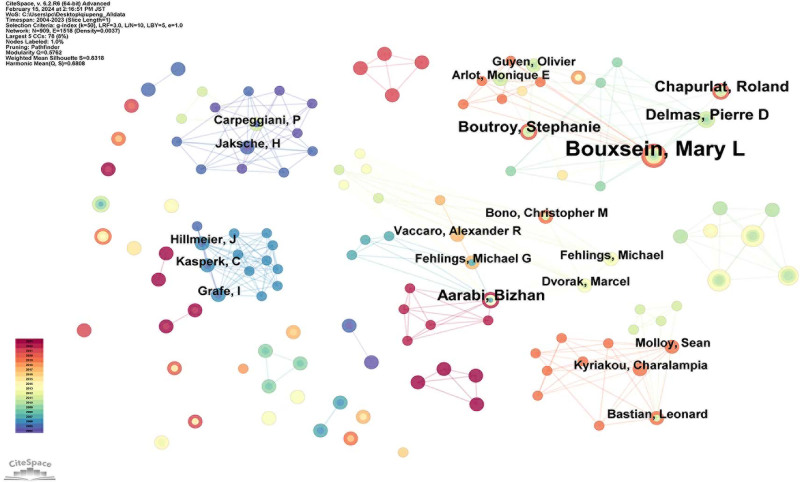
Map of authors related to AI for VCF. *Note*: Each node represents an author, with a node size indicating their citation count. The lines between authors represent collaborative relationships. AI = artificial intelligence, VCF = vertebral compression fractures.

In the domain of AI applications for VCF, 909 authors have contributed to the publication of materials, forming a collaborative network, as illustrated in Figure 6. The top 10 authors by number of publications are listed in Table 3, in descending order: Bouxsein, Mary L (6 publications), Biffar, Andreas (5 publications), Baur-Melnyk, Andrea (5 publications), Dietrich, Olaf (4 publications), Aarabi, Bizhan (3 publications), Kariya, Shuji (3 publications), Boutroy, Stephanie (3 publications), Dreher, Maureen L (3 publications), Delmas, Pierre D (3 publications), and Chapurlat, Roland (3 publications). Among these, Aarabi and Bizhan are the most influential authors in the field. For instance, in 2013, this author developed a widely accepted, comprehensive, yet simple VCF classification system using AI, which has been cited 456 times.^[33]^

**Table 3:** Top 10 authors related to artificial intelligence for vertebral compression fractures.

Rank	Author	Frequency	Year	Country
1	Bouxsein, Mary L	6	2009	USA
2	Biffar, Andreas	5	2010	Germany
3	Baur-melnyk, Andrea	5	2010	Germany
4	Dietrich, Olaf	4	2010	Germany
5	Aarabi, Bizhan	3	2007	USA
6	Kariya, Shuji	3	2007	Japan
7	Boutroy, Stephanie	3	2010	France
8	Dreher, Maureen L	3	2013	USA
9	Delmas, Pierre D	3	2009	France
10	Chapurlat, Roland	3	2010	France

### 3.4. Analysis of countries and institutions

**Figure 7.**
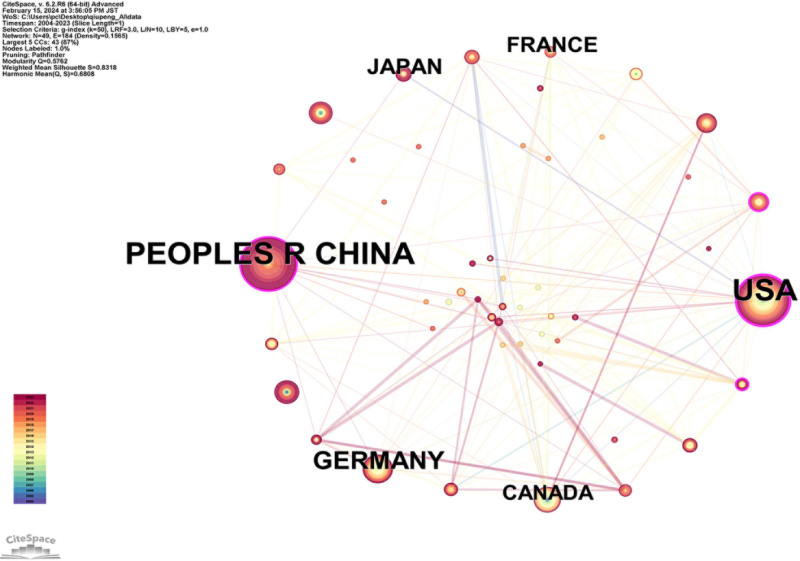
Map of countries related to AI for VCF. *Note*: The node size is proportional to the volume of publications. Lines between countries represent collaborative relationships. AI = artificial intelligence, VCF = vertebral compression fractures.

Figure 7 depicts a network diagram of country collaborations consisting of 49 countries and 184 connections. Table 4 shows the top 10 countries ranked by the number of publications and centrality. It was found that the United States and China had the highest volume of publications in the field of AI applications in VCF, holding top positions in global influence. This correlation is not only with the countries’ technological levels but also related to the base population affected by VCF. Although countries with less advanced technology have fewer publications in this field, the high prevalence of VCF in these countries should not be overlooked.

**Table 4:** Top 10 frequency and centrality of countries related to artificial intelligence for vertebral compression fractures.

Rank	Frequency	Countries	Rank	Centrality	Countries
1	134	Peoples R China	1	0.35	USA
2	129	USA	2	0.14	Peoples R China
3	50	Germany	3	0.12	Belgium
4	28	Japan	4	0.11	England
5	27	France	5	0.09	Italy
6	25	Canada	6	0.07	France
7	23	South Korea	7	0.06	Canada
8	22	Taiwan	8	0.06	Switzerland
9	19	Switzerland	9	0.05	Sweden
10	16	England	10	0.04	Germany

**Figure 8.**
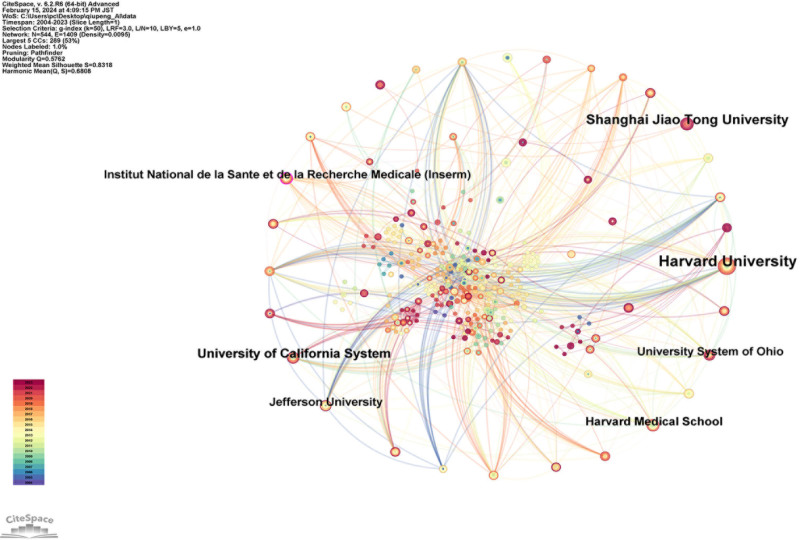
Map of institutions related to AI for VCF. *Note*: The node size is proportional to the volume of publications. The lines between the institutions represent collaborative relationships. AI = artificial intelligence, VCF = vertebral compression fractures.

Furthermore, a visual analysis of the academic institutions corresponding to these countries was conducted (Fig. 8), and the top 10 academic institutions by number of publications are listed in Table 5, in descending order: Harvard University (19 publications), Shanghai Jiao Tong University (13 publications), University of California System (11 publications), Harvard Medical School (9 publications), Institut National de la Santé et de la Recherche Médicale (Inserm) (8 publications), University System of Ohio (8 publications), Jefferson University (8 publications), Beth Israel Deaconess Medical Center (7 publications), Assistance Publique Hôpitaux Paris (APHP) (7 publications), and Humboldt University of Berlin (7 publications).

**Table 5:** Top 10 publications of institutions related to artificial intelligence for vertebral compression fractures.

Rank	Frequency	Year	Institutions
1	19	2005	Harvard University
2	13	2013	Shanghai Jiao Tong University
3	11	2007	University of California System
4	9	2009	Harvard Medical School
5	8	2010	Institut National de la Sante et de la Recherche Medicale (Inserm)
6	8	2009	University System of Ohio
7	8	2005	Jefferson University
8	7	2010	Beth Israel Deaconess Medical Center
9	7	2009	Assistance Publique Hopitaux Paris (APHP)
10	7	2012	Humboldt University of Berlin

### 3.5. Analysis of cited journals

**Figure 9.**
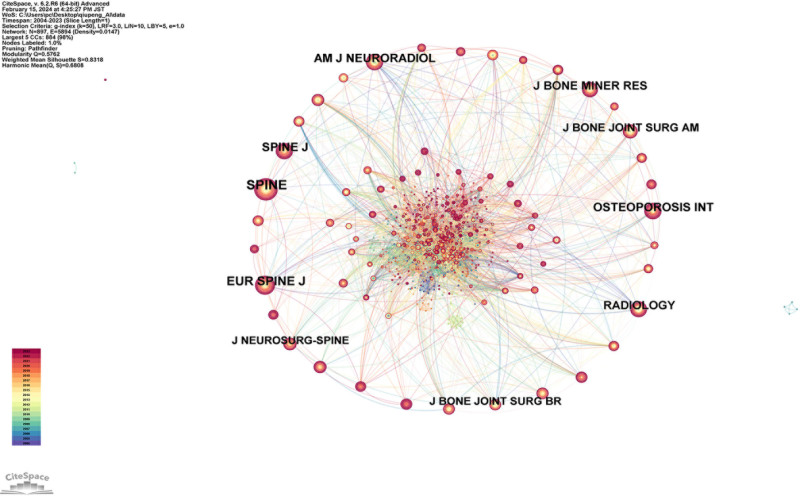
Map of cited journals related to AI for VCF. *Note*: The node size is proportional to the volume of publications. The lines between journals represent collaborative relationships. AI = artificial intelligence, VCF = vertebral compression fractures.

A total of 897 journals have been cited in the realm of AI applications for VCF, with these journals interconnected by approximately 5894 links. The cited journals are depicted in the cited journal network diagram (Fig. 9). In addition, the top 10 journals ranked by the number of citations are listed in Table 6, in descending order: SPINE (322 citations), EUR SPINE J (235 citations), SPINE J (177 citations), OSTEOPOROSIS INT (174 citations), AM J NEURORADIOL (173 citations), J BONE MINER RES, RADIOLOGY (167 citations), J BONE JOINT SURG AM (134 citations), J BONE JOINT SURG BR (109 citations), and JNEUROSURG-SPINE (107 citations). SPINE, a journal specializing in orthopedics and clinical neurology, is the most influential publication in this field.

**Table 6:** Top 10 frequency of cited journals related to artificial intelligence for vertebral compression fractures.

Rank	Frequency	Year	Cited journals
1	322	2004	SPINE
2	235	2004	EUR SPINE J
3	177	2007	SPINE J
4	174	2005	OSTEOPOROSIS INT
5	173	2004	AM J NEURORADIOL
6	168	2004	J BONE MINER RES
7	167	2004	RADIOLOGY
8	134	2005	J BONE JOINT SURG AM
9	109	2004	J BONE JOINT SURG BR
10	107	2007	J NEUROSURG-SPINE

## 4. Discussion

### 4.1. General information

This study systematically explored and visualized the citation landscape of AI applications in VCF research over the past 2 decades. While publication volume remained modest before 2018, 2019 marked a pivotal inflection point characterized by a sharp increase in research output. This surge coincided with the maturation and clinical integration of key AI technologies, particularly DL, underscoring the strong link between technological innovation and scholarly attention. Previous studies have similarly emphasized how breakthroughs in computational techniques often precede or accompany rapid expansions in biomedical research domains, and our findings reinforce this trajectory within the VCF field.

One notable driver of this shift is the introduction of iterative fully convolutional neural networks for automated vertebra segmentation and diagnosis, technologies that improve analytical precision and reduce the manual burden in radiological workflows. These advances exemplify the broader trend in AI transitioning from a theoretical concept to a clinically applicable tool. This transition has not only enhanced research efficiency, but has also sparked new lines of inquiry, such as real-time image interpretation, AI-assisted prognosis, and personalized surgical planning.

Our keyword and timeline analysis revealed a marked thematic evolution: from traditional procedural terms like “vertebroplasty” and “augmentation” toward algorithmic and data-driven concepts such as “deep learning” and “convolutional neural networks.” This reflects a paradigm shift in how VCF is approached, from a mechanical treatment-centered model to a more data-informed diagnostic-optimization model. Interestingly, this evolution parallels developments in other specialties, suggesting that AI adoption in VCF may serve as a microcosm for broader medical transformation trends.

In addition to thematic changes, our collaboration network analysis highlights the growing importance of interdisciplinary and international cooperation. The complexity of applying AI to VCF, which requires expertise in orthopedics, computer science, radiology, and biomedical engineering, has naturally fostered a more interconnected research community. However, disparities remain. Our findings suggest that research output is geographically concentrated, with China and the United States leading the field. This raises important questions about equitable access to AI infrastructure and the potential underrepresentation of low-resource settings in shaping global research agenda.

Furthermore, the role of academic institutions and journals as amplifiers of innovation cannot be overlooked. Our analysis identified the concentration of output and influence within a small number of high-impact journals and elite research centers. While this may streamline knowledge dissemination, it also risks gatekeeping, in which emerging researchers or novel approaches outside traditional academic hubs struggle for visibility. Addressing this imbalance may require more open-access publication models, cross-institutional data-sharing platforms, and funding mechanisms to promote global inclusivity.

Taken together, these findings reveal not only the rapid evolution of AI-driven research in VCF, but also the underlying ecosystem of collaboration, institutional power, and thematic realignment that propels it. Future investigations should explore how AI tools can be equitably deployed across diverse healthcare systems, and how interdisciplinary structures can be institutionalized to sustain innovation. More critically, attention should be given to translating technical breakthroughs into clinically validated outcomes, ensuring that AI’s promise does not remain confined to the research arena, but meaningfully impacts patient care.

### 4.2. Research hotspots

Building on the findings of our keyword analysis and citation burst detection, it is evident that the integration of AI, particularly DL, has played a transformative role in the domain of VCF diagnosis and treatment. Two primary research hotspots have emerged: first, the application of diverse AI algorithms, especially DL models, in the interpretation of multimodal imaging data for the accurate and early diagnosis of VCF. Second, the growing clinical prospects of robot-assisted surgery, which enhance surgical precision and personalization in VCF treatment. These trends reflect a paradigm shift driven by technological advancements and are consistent with the sharp increase in publication volume since 2019.

### 4.3. Capability of different AI algorithms in processing VCF diagnosis

The application of DL in the analysis of X-ray, CT, and MRI images for VCF signifies a major advancement in the diagnostic methods. Several studies^[34–38]^ have utilized DL algorithms to identify X-ray data of patients with VCF, achieving classification of the nature of the disease. In the radiological data from CT, Choi et al^[39]^ and Zhang et al^[40]^ employed weakly supervised DL to enhance the sensitivity of diagnosing multiple vertebral compression fractures, and Duan et al^[41]^ used the same approach to differentiate between benign and malignant VCFs. Several studies have employed AI to perform high-performance differential diagnoses between benign and malignant VCFs using MRI data, which are sensitive to soft tissue signals.^[42–44]^

Among AI algorithms, Convolutional Neural Networks (CNN) are the most widely applied, with numerous studies leveraging CNN to significantly increase the accuracy in diagnosing VCF.^[45–47]^ Compared to traditional algorithms, CNNs can better handle high-dimensional, complex data, especially demonstrating powerful performance in the analysis of VCF images and videos.^[48,49]^ Additionally, algorithms like Support Vector Machines, Logistic Regression, Gradient Boosting Machines, Extreme Gradient Boosting, Random Forest, Decision Trees, and Multilayer Perceptrons are continuously being updated and validated.^[50–53]^ AI technology is superior in processing and analyzing multimodal imaging data, allowing for a more comprehensive evaluation of VCF through DL models by integrating different types of imaging information, such as X-rays, CT, and MRI. This integrated analysis not only improves diagnostic accuracy but also opens possibilities for the early diagnosis and treatment of the disease.

### 4.4. Prospects of robotic-assisted surgery in treating VCF

With the integration of AI and robotics technology, robot-assisted surgery has emerged as a new direction in the treatment of VCF. The application of robotic technology enables precise surgical positioning, allowing minimally invasive surgery through smaller incisions, which not only reduces the postoperative recovery time but also enhances surgical precision. Moreover, robot-assisted surgery can formulate personalized treatment plans based on specific patient conditions, further improving treatment outcomes and safety. Several studies utilizing robot-assisted surgery have demonstrated satisfactory results in terms of precision.^[54–56]^ Additionally, investigations into patient clinical questionnaire scales by Liu et al^[57]^ and Qian et al^[58]^ have shown that patients recover better following robot-assisted surgery.

## 5. Conclusion

In conclusion, this BA revealed that the application of AI in VCF has transitioned from a focus on surgical interventions to a new era driven by DL. Our analysis mapped the field’s intellectual structure, identifying a critical “translation gap” between algorithm development and clinical outcome validation. Furthermore, we characterized a bipolar research landscape dominated by the US and China, suggesting opportunities for enhanced international collaboration. These findings provide a strategic roadmap for researchers, clinicians, and policymakers to navigate this promising field.

## 6. Limitations

This study has several limitations inherent to the bibliometric methodology. First, relying solely on the WoSCC may omit relevant studies from other databases such as PubMed or Scopus, potentially affecting data completeness. Second, owing to the rapid evolution of AI, particularly DL, bibliometric analyses may struggle to reflect real-time clinical applications, as publication often lags behind innovation. Third, citation-based metrics do not account for study quality, treat high- and low-quality research equally, and may overlook novel but under-cited work. Integrating bibliometric analyses with systematic reviews, altmetrics, or expert-informed appraisal frameworks may offer a more comprehensive view. Finally, while bibliometrics excels at mapping “what” trends exist, it cannot explain “why” they emerge: such as the specific drivers behind the 2019 publication surge. These underlying causes likely involve complex factors, such as funding shifts, policy incentives, or technological breakthroughs outside the scope of citation data. Future research should consider complementary qualitative methods to uncover these deeper drivers and contextualize bibliometric findings.

## Acknowledgments

We extend our gratitude to Chaomei Chen of Drexel University for his contribution in developing CiteSpace. We would also like to express our appreciation to the reviewers for their valuable insights that have enabled us to enhance this manuscript.

## Author contributions

**Conceptualization:** Xue-Feng Ma, Hang Ren.

**Data curation:** Xue-Feng Ma.

**Formal analysis:** Peng Qiu, Hang Ren.

**Investigation:** Peng Qiu.

**Project administration:** Peng Qiu.

**Methodology:** Dong-Xia Chen.

**Software:** Dong-Xia Chen, Hang Ren.

**Supervision:** Hang Ren.

**Validation:** Dong-Xia Chen.
